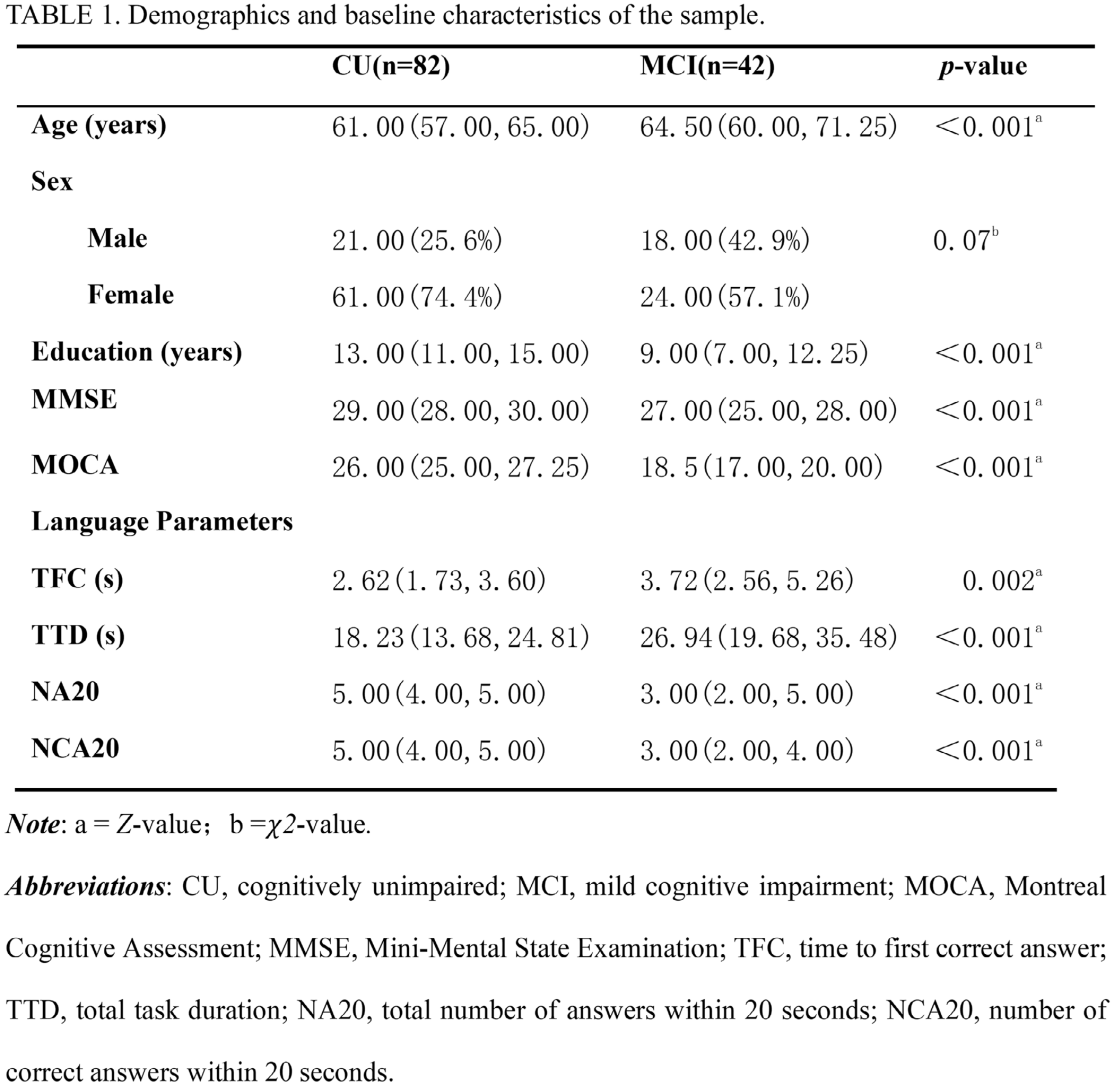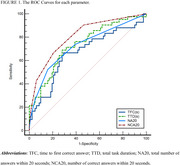# Specific Parameters of the Serial 7s Task with Audio Recording: Potential Early Self‐screening Biomarkers for Mild Cognitive Impairment

**DOI:** 10.1002/alz70856_103929

**Published:** 2025-12-26

**Authors:** Lin Hu, Xiang Fan, Keyan Yu, Yining Liao, Lele Chen, Zhuonan Wei, Gaigai Lu, Hui Chen, Tong Wu, Silin Tao, Guanxun Cheng

**Affiliations:** ^1^ Peking University Shenzhen Hospital, Shenzhen, Guangdong, China; ^2^ The Chinese University of Hong Kong – Shenzhen, Shenzhen, Guangdong, China

## Abstract

**Background:**

Mild Cognitive Impairment (MCI) is a clinical staging between cognitively unimpaired (CU) and dementia. Promoting a fast, simple, effective, and instrument‐free self‐screening test is essential for achieving early diagnosis and timely intervention. Studies indicate that individuals in the MCI stage exhibit significantly prolonged mean reaction times, reduced accuracy in working memory tasks, and decreased attentional resource allocation in dual‐task scenarios. The Serial 7s task, a widely used cognitive assessment tool, evaluates working memory, attention, and information processing speed. Performance on this task has been shown to correlate with the severity of cognitive impairment in MCI patients. Research on the Japanese version of the Mini‐Mental State Examination (MMSE‐J) demonstrated that the Serial 7s task achieves a sensitivity of 0.86 and a specificity of 0.89 for detecting MCI, with a test‐retest reliability of 0.81. However, existing research mainly focuses on the association between this task performance and cognitive impairment, and specific parameters have been limitedly listed. Thus, this study aimed to evaluate the potential of the specific parameters of Serial 7s task parameters as early self‐screening biomarkers for MCI.

**Method:**

The study included 42 MCI patients and 82 CU individuals with audio recordings of the Serial 7s task from the Shenzhen mulTi‐modal Aging Research (STAR) cohort. The Audio recordings of the Serial 7s task were analyzed to extract the following specific parameters: time to first correct answer (TFC), total task duration (TTD), total number of answers within 20 seconds (NA20), and number of correct answers within 20 seconds (NCA20). Statistical analyses were conducted using SPSS version 27.0.

**Result:**

All task parameters showed significant differences between CU and MCI individuals (*p* < 0.05). Among these, NCA20 demonstrated the best diagnostic performance, achieving the highest accuracy with an area under the curve (AUC) of 0.81 (95% CI: 0.728–0.873).

**Conclusion:**

The specific parameters derived from the Serial 7s task with audio recording, particularly NCA20, show potential as early self‐screening biomarkers for MCI due to their convenience and efficiency. Future research with larger sample sizes is needed to validate these findings and further refine their diagnostic utility.